# PEPOP: Computational design of immunogenic peptides

**DOI:** 10.1186/1471-2105-9-71

**Published:** 2008-01-30

**Authors:** Violaine Moreau, Cécile Fleury, Dominique Piquer, Christophe Nguyen, Nicolas Novali, Sylvie Villard, Daniel Laune, Claude Granier, Franck Molina

**Affiliations:** 1 CNRS FRE 3009, SysDiag, CAP DELTA, 1682 Rue de la Valsière, CS 61003, 34184 Montpellier Cedex 4, France

## Abstract

**Background:**

Most methods available to predict protein epitopes are sequence based. There is a need for methods using 3D information for prediction of discontinuous epitopes and derived immunogenic peptides.

**Results:**

PEPOP uses the 3D coordinates of a protein both to predict clusters of surface accessible segments that might correspond to epitopes and to design peptides to be used to raise antibodies that target the cognate antigen at specific sites. To verify the ability of PEPOP to identify epitopes, 13 crystallographically defined epitopes were compared with PEPOP clusters: specificity ranged from 0.75 to 1.00, sensitivity from 0.33 to 1.00, and the positive predictive value from 0.19 to 0.89. Comparison of these results with those obtained with two other prediction algorithms showed comparable specificity and slightly better sensitivity and PPV. To prove the capacity of PEPOP to predict immunogenic peptides that induce protein cross-reactive antibodies, several peptides were designed from the 3D structure of model antigens (IA-2, TPO, and IL8) and chemically synthesized. The reactivity of the resulting anti-peptides antibodies with the cognate antigens was measured. In 80% of the cases (four out of five peptides), the flanking protein sequence process (sequence-based) of PEPOP successfully proposed peptides that elicited antibodies cross-reacting with the parent proteins. Polyclonal antibodies raised against peptides designed from amino acids which are spatially close in the protein, but separated in the sequence, could also be obtained, although they were much less reactive. The capacity of PEPOP to design immunogenic peptides that induce antibodies suitable for a sandwich capture assay was also demonstrated.

**Conclusion:**

PEPOP has the potential to guide experimentalists that want to localize an epitope or design immunogenic peptides for raising antibodies which target proteins at specific sites. More successful predictions of immunogenic peptides were obtained when a peptide was continuous as compared with peptides corresponding to discontinuous epitopes. PEPOP is available for use at .

## Background

In antibody-antigen (Ab-Ag) interactions, the paratope of the Ab binds to the epitope of the Ag. The identification of epitopes is an important step for understanding molecular recognition rules and is also helpful for diagnosis of diseases and for drug and vaccine design. The ultimate method to precisely define an epitope is to solve the 3D structure of the Ab-Ag complex either by X-ray crystallography or NMR [1]. These techniques are, however, demanding and generally time-consuming. Faster epitope identification methods have been described such as site-directed mutagenesis of the Ag [2,3]. Another popular approach to map an epitope is parallel peptide synthesis [4,5], based on the synthesis of overlapping peptides covering the entire Ag sequence. In this case, mainly continuous (sequential or linear) epitopes can be identified. Screening chemical or biological combinatorial libraries [6] for Ab binders allows selection of peptides also called mimotopes [7], mimicking more or less faithfully the epitope. Bioinformatics tools have been developed to help experimentalists in localizing the epitope by the sequence analysis of the selected mimotopes [8,9].

Synthetic peptides are commonly used as immunogens to raise anti-peptide Abs that may cross-react with proteins [10], thus allowing their detection and quantification. These peptides are generally designed by using methods that attempt to predict antigenic determinants of a protein. Numerous algorithms have been developed over the past 25 years. They are based on different theoretical physicochemical characteristics of the target protein such as hydrophilicity, flexibility, accessibility, and secondary structure, especially turns [11]. Other methods are combinations of the latter approaches [12], the most recent [13] being an extension and combination of the methods of Parker *et al*. [14] and Jameson and Wolf [15]. Likewise, Welling *et al*. [16] developed an antigenicity scale, with the aim of predicting antigenic regions and synthesizing the corresponding antigenic peptides to elicit Abs reactive with the intact protein. All these algorithms have led to the development of several softwares or web interfaces that make the use of such methods very easy. It is, however, difficult to assess the efficacy of all predictive methods. A comparative study published some years ago [11,17] indicated that the most accurate predictive method at that time is based on the prediction of turns. This method was implemented in BEPITOPE [18]. A more recent and more exhaustive comparative study [19] concluded that the methods based on sequence analysis do not predict epitopes better than chance.

All these methods predict antigenic determinants from the protein sequence alone, neglecting 3D structure information. This is surprising because the 3D structure of an increasing number of proteins has been solved by X-ray crystallography or NMR, and predictive modeling methods are available that show increasing accuracy [20]. Recently, however, a few recent studies [21-24] propose bioinformatics tools based on 3D information to predict epitopes.

In this article, we describe PEPOP, an algorithm that makes use of the 3D information of a protein to predict peptides which could serve as immunogens to raise site-specific anti-protein Abs. Clusters of surface accessible segments of the protein are first identified by PEPOP, and this information is further used to design the peptides. We analyzed how PEPOP clusters corresponded to structurally defined epitopes (dataset of 13 epitopes on 8 antigens) and how Abs raised against peptides designed by PEPOP reacted with the parent protein.

## Results

### Clustering of exposed segments of the Ag

#### A- PEPOP features and outputs for clustering

**Figure 1:**
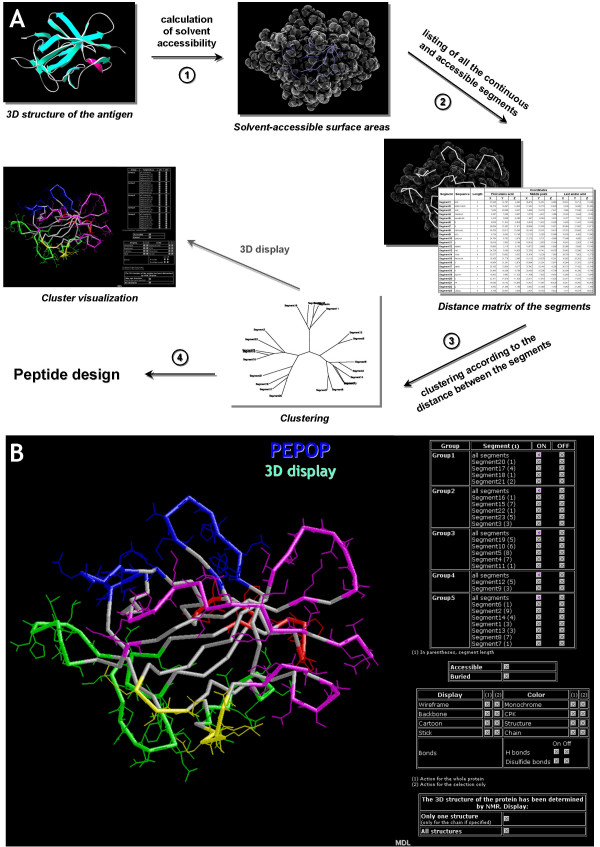
**PEPOP**. **(A) **Description of PEPOP. Step 1: The solvent accessible surface area is calculated from the 3D structure of a protein. Step 2: Segments of accessible and contiguous amino acids are listed, and the shortest Euclidian distance matrix between segments is calculated. Step 3: The segments are clustered according to the matrix (clusters can be displayed on the 3D structure of the Ag). Step 4: Peptides are designed according to the clustering analyses. **(B) **The PEPOP interface. Segments and clusters can interactively be displayed on a 3D view of the Ag.

We developed the PEPOP algorithm as a new method intended to identify peptide sequences that, when injected into animals, induce the production of Abs that should recognize specific areas of a protein. From the 3D structure of a protein, PEPOP first identifies segments composed of accessible and sequence contiguous amino acids. Then, these segments are clustered according to their spatial distances (Figure 1A). Clusters and their segments are then further used to design immunogenic peptides. The PEPOP interface was designed so as to provide both detailed information (atomic coordinates, distance matrix, etc.) and modifiable views of the cluster(s) in the 3D context of the protein (Figure 1B).

#### B- Experimental evaluation of PEPOP capacity to predict antigenic epitopes

Since there is a documented relationship between surface accessibility and antigenicity [25,26], we first evaluated the correspondence between the surface clusters identified by the PEPOP algorithm on given proteins and their known epitopes. To this end, 13 Ab-Ag complexes for which the amino acids composing the discontinuous epitope have been identified precisely were studied (selected from the literature and from the PDB [27]). A detailed analysis was first carried out on hen egg lysozyme (HEL) because several structurally, well-defined epitopes have been identified (Table 1) by X-ray crystallography (resolutions from 1.5 to 3.22 Å). The PEPOP algorithm was run on the 3D structure of HEL [PDB: 1SFG]. PEPOP identified 23 segments of accessible residues which were automatically grouped into four clusters on the surface of HEL (Table 1). Cluster 1 contains antigenic residues belonging to three epitopes (D11.15, HyHEL-10, and HyHEL-63). Cluster 2 exactly matches the D1.3 epitope and 9 of 10 residues of the D11.15 epitope; it also predicts some of the antigenic residues of the HyHEL-10 and HyHEL-63 epitopes. Cluster 3 perfectly matches the D44.1 epitope and almost perfectly the HyHEL-5 epitope (13 of 14 residues); it also predicts part of the HyHEL-10 and HyHEL-63 epitopes. Cluster 4 does not correspond to any X-ray described epitope of the lysozyme.

**Table 1:** Distribution of the residues of the HEL epitopes in the clusters identified by PEPOP

PEPOP cluster	PEPOP segment	Position ([PDB: 1SFG] chain A)	mAb^1^					
			D1.3	D11.15	D44.1	HyHEL-5	HyHEL-10	HyHEL-63
Cluster1	Segment17	93–94		**1/10 **(103)			**7/16 **(93, 96, 97, 100, 101, 102, 103)	**7/23 **(93, 96, 97, 100, 101, 102, 103)
	Segment18	96–97						
	Segment19	100–103						
Cluster2	Segment2	10–16	**10/10 **(13, 14, 19, 21, 22, 24, 117, 119, 121, 125)	**9/10 **(21, 23, 106, 112, 113, 116, 117, 118, 119)			**5/16 **(15, 16, 19, 20, 21)	**8/23 **(13, 14, 15, 16, 18, 19, 20, 21)
	Segment20	106–109						
	Segment21	111–114						
	Segment22	116–119						
	Segment23	121–129						
	Segment3	18–24						
	Segment4	27–28						
Cluster3	Segment10	56–57			**11/11 **(41, 45, 46, 47, 49, 53, 67, 68, 70, 81, 84)	**13/14 **(41, 43, 44, 45, 46, 47, (63, 73, 75, 48, 49, 89) 53, 67, 68, 70, 84)	**4/16**(63, 73, 75, 89)	**6/23 **(62, 63, 73, 75, 77, 89)
	Segment11	59						
	Segment12	61–63						
	Segment13	65–68						
	Segment14	70–79						
	Segment15	81–82						
	Segment16	84–90						
	Segment8	41–49						
	Segment9	53						
Cluster4	Segment1	1–8						
	Segment5	33–35						
	Segment6	37						
	Segment7	39						
amino acids not accessible						**1/14 **(69)		**2/23 **(98, 99)

^1 ^Ratio of the number of amino acids in the epitope included in the cluster to the total number of amino acids in the epitope. The epitopes were defined as in the original publication (D1.3 [54], D11.15 [55], D44.1 [56], HyHEL-5 [57], HyHEL-10 [58], HyHEL-63 [59]). Positions of the amino acids in the epitope included in the cluster are indicated in parentheses. The ratios are in bold for the major clusters.

**Figure 2:**
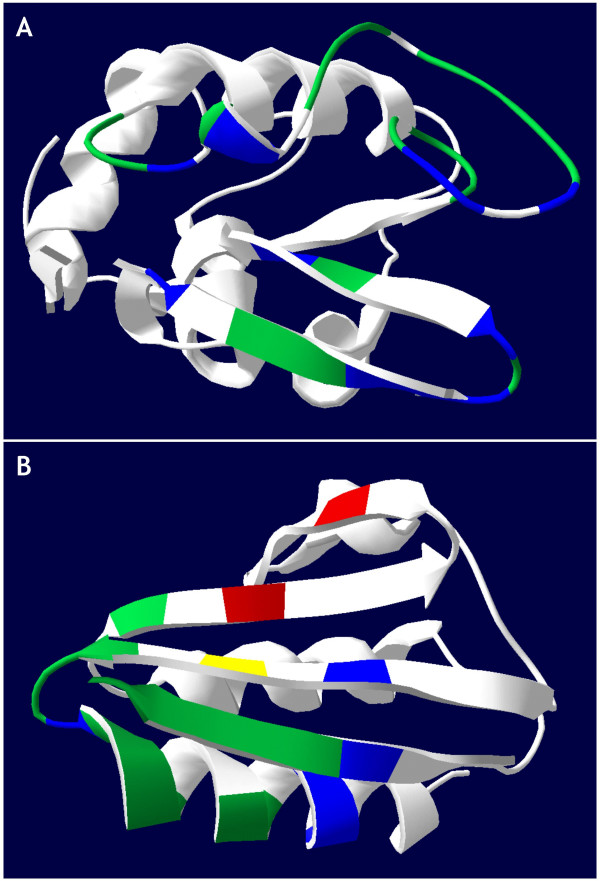
**Comparison of known epitopes with the predicted major cluster**. Amino acids in the epitope and in the major cluster are in green; those outside of the major cluster are in red; those non-accessible are in yellow; amino acids in the major cluster but not in the epitope are in blue. **(A) **Epitope on HEL recognized by mAb D44.1 **(B) **Epitope on HPr recognized by mAb Jel42.

Next, seven additional Ab-Ag complexes were analyzed to extend these observations to a database of 13 crystallographic epitopes (Table 2). PEPOP analyses were performed on the 3D structures of the Ag alone, i.e., not complexed with the mAb specific for the epitope studied because this is the most frequently encountered case. The distribution of the amino acids in the epitope among the clusters identified by PEPOP was analyzed, and the number of the amino acids in the epitope in the major cluster (cluster containing the greatest number of amino acids of the epitope) was calculated. The results show that the experimentally identified amino acids in the epitope belong to a single cluster for the D1.3, D44.1, and HyHEL-5 epitopes in HEL and the 5G9 epitope in tissue factor, to two clusters for the D11.15 epitope in HEL and the Jel42 epitope in the histidine-containing phosphocarrier protein HPr or to three clusters for the complexes BH151 – hemagglutinin, Bo2C11 – C2 domain of FVIII, NC41 – neuraminidase, F9.13.7 – guinea fowl lysozyme (GEL), HyHEL-10 – HEL, HyHEL-63 – HEL, and N10 – staphylococcal nuclease (SN). For example, all the amino acids of the epitope on HEL recognized by mAb D44.1 are included in a single cluster identified by PEPOP (Figure 2A), and 13 amino acids out of 16 of the epitope on HPr recognized by mAb Jel42 are included in the major cluster (Figure 2B). Table 2 shows that the specificity of the method ranged from 0.75 to 1.00 (median value 0.87) and the sensitivity varied within a broader range (0.33 to 1.00; median value: 0.63). The positive predictive value (PPV) varied from 0.19 to 0.89 (mean value 0.43; median value 0.33). The same database of 13 crystallographically defined epitopes was used with two freely available web tools that also make use of the 3D information of the protein to predict epitopes (DiscoTope [23,28] and CEP [24,29]). The results in Table 2 show that both methods have similar specificity (median value for DiscoTope: 0.83; median value for CEP: 0.90). Their sensitivities, however, are slightly lower than that of PEPOP (median value for DiscoTope: 0.39; median value for CEP: 0.47) and the PPV are again lower than that found for PEPOP predictions (median value for DiscoTope: 0.17; median value for CEP: 0.28) (Table 2). Thus, performances of PEPOP compare well with similar, but not identical, algorithms.

**Table 2:** Evaluation and comparison of the performances of PEPOP

Ab – Ag complex^1^	PDB	Epitope (number of amino acids)	PEPOP					CEP			DiscoTope		
			
			Number of clusters containing epitopic residues/total number of clusters	Number of predicted residues of the epitope	Sp	Se	PPV	Sp	Se	PPV	Sp	Se	PPV
D1.3 – hen egg lysozyme [54]	1SFG_A	10	1/4	10	0.77	1.00	0.27	0.76	1.00	0.26	0.80	0.40	0.14
D44.1 – hen egg lysozyme [56]	1T6V_M	11	1/5	11	0.81	1.00	0.33	0.78	0.91	0.28	0.86	0.55	0.27
HyHEL-5 – hen egg lysozyme [57]	1VDP_B	14	1/5	13	0.83	0.93	0.41	0.88	0.00	0.00	0.78	0.00	0.00
Jel42 – HPr [60]	1POH	16	2/8	13	0.93	0.81	0.72	0.90	0.75	0.63	0.97	0.06	0.33
D11.15 – hen egg lysozyme [55]	1HEL	10	2/5	8	0.95	0.80	0.57	0.90	0.10	0.08	0.82	0.30	0.13
5G9 – tissue factor [61]	1WV7_T	18	1/3	14	0.75	0.78	0.25	0.84	0.28	0.15	0.84	0.17	0.10
BH151 – hemagglutinin [62]	5HMG_C	19	3/7	12	0.83	0.63	0.19	0.97	0.32	0.40	0.75	0.11	0.03
Bo2C11 – FVIII C2 domain [63]	1D7P_M	15	3/5	8	0.92	0.53	0.42	0.94	0.47	0.44	0.83	0.53	0.25
NC41 – neuraminidase [64]	1NMC_N	22	3/4	11	0.90	0.50	0.23	0.99	0.59	0.81	0.85	0.64	0.20
F9.13.7 – guinea fowl lysozyme [65]	1HHL	10	3/5	5	0.98	0.50	0.71	0.92	0.50	0.33	0.83	0.40	0.17
HyHEL-10 – hen egg lysozyme [58]	1UC0_A	16	3/5	8	0.99	0.50	0.89	0.96	0.56	0.64	0.87	0.44	0.32
HyHEL-63 – hen egg lysozyme [59]	1VFB_C	23	3/4	9	0.82	0.39	0.32	0.73	0.30	0.19	0.87	0.39	0.39
N10 – staphylococcal nuclease [66]	1EYO_A	18	3/6	6	0.87	0.33	0.29	0.88	0.06	0.07	0.66	0.33	0.13

**Average**					**0.87**	**0.67**	**0.43**	**0.88**	**0.45**	**0.33**	**0.83**	**0.33**	**0.19**
**Median**					**0.87**	**0.63**	**0.33**	**0.90**	**0.47**	**0.28**	**0.83**	**0.39**	**0.17**

^1 ^The corresponding reference is given in parentheses

nd: not determined (no results returned from the server)

Sp: specificity; Se: sensibility; PPV: positive predictive value (see Materials and methods)

### Design of peptides from clusters

#### A- PEPOP features and outputs for the design of immunogenic peptides

**Figure 3:**
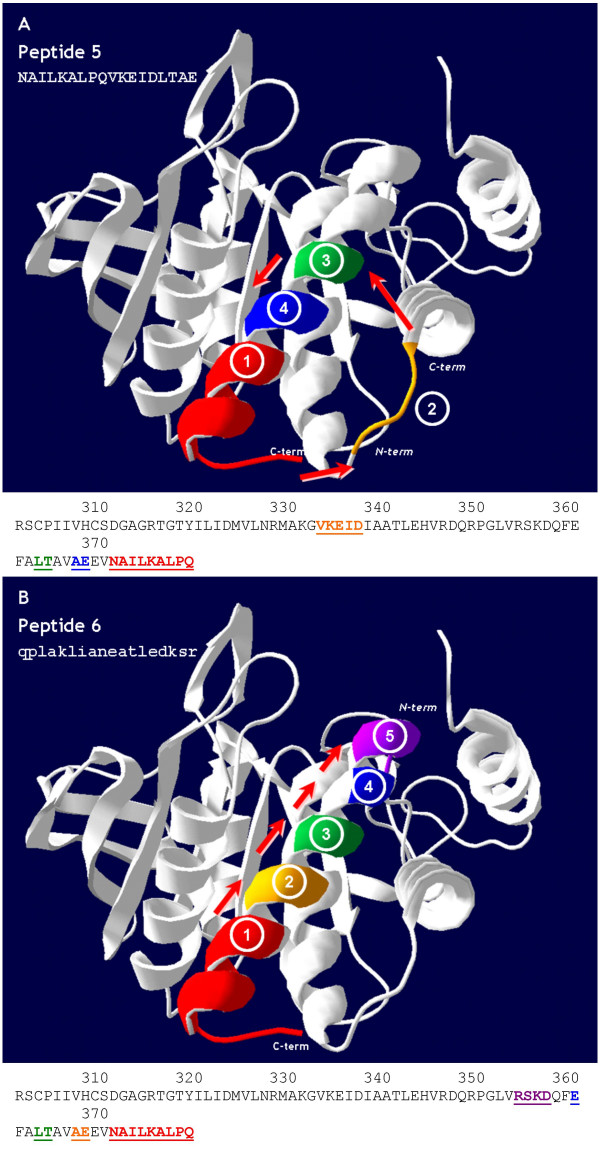
**Example of peptides designed from the 3D structure of IA-2**. The two peptides, #5 (A) and #6 (B) of Table 3, were constructed from the longest segment (segment 1: reference segment) to which were added the nearest neighbor segments (segment 2 to respectively segment 4 and segment 5), according to two different methods (respectively method NN and NNd). Segments indicated in the IA-2 sequence are in the same color as in the 3D structure.

Based on PEPOP prediction of clusters of surface accessible segments, the design of peptides suitable for raising Abs potentially cross-reactive with the target protein can be achieved by different methods. To build *in silico *a candidate peptide, a segment, called a "reference segment", is first chosen, and its sequence is extended with a "method of extension" in a specified "area of extension" on the protein to yield the designed peptide. Each of the reference segments, the method, and the area of extension can be selected among several possibilities. Figure 3 illustrates the method of adding the nearest neighbor (NN) segments to the reference segment to obtain a suitable peptide. Peptides #5 and #6 in Table 3 were constructed from the longest segment to which the nearest neighbor segments were added, according to two different methods (respectively, NN and NNd, for segments synthesized using D-amino acids). The software was developed in such a way that, at each of the three steps of the design of a given peptide, the user can choose the parameter (for example, inclusion or not of a part of the protein in the peptide) or let the algorithm automatically do it. By default, the reference segment is the longest segment, the method of extension is the addition of the protein sequence and in this case it is not necessary to select an area of extension. The peptide is therefore extended until the default minimal length of 20 amino acids is reached.

**Table 3:** Reactivity with the cognate Ag of the Abs obtained by immunization with the peptides

			Method^2 ^			
Ag	Peptide #	Peptide sequence^1^	Reference segment^3^	Method of extension^4^	Area of extension^5^	A_450 nm _for serum diluted 1:10 000^6^
IA-2	1	VSSQFSDAAQASPS		B		1.75
	2	SPSSHSSTPSWCEEP		B		0.30
	3	RYWPDEGASLYHVYEV		B		0.36
	4	ALTAVAEEVNAILKALPQ	L	FPS	-	3.62
	5	NAILKALPQVKEIDLTAE	L	NN	CC	0.14
	6	qplaklianeatledksr	L	NNd	CC	0.02
	7	diekvgkaGPGNAILKALPQ	L	MD	CC	0.02
	8	ARIKLKVESSPSRSDYIN	T	FPS	SC	1.83
TPO	9	VVTDDDRYSDLLMAWGQ		B		3.76
	10	ARLRDSGRAYLPFVPP		B		0.46
	11	PYEGYDSTANPTVSNVF		B		0.06
	12	EDFESCDSIPGMNLEA		B		0.39
	13	RRLDASFQEHPDLPGLWLH	L	FPS	-	3.75
	14	ASFQEHPDLPGRLWQFVLS	L	NN	CC	0.33
	15	APEPGIPGERPCPPRAPAA	T	NN	TC	0.47
	16	ASFQEHPDLPGRAPEPGIPGE	L	MD	CC	0.14
IL8	17	PKFIKELRVIESGPHCANT	L	FPS	-	0.16
	18	ENWVQRVVEKFLKRAENS	T	FPS	SC	2.25

^1 ^Amino acids in lower case are D-amino acids. ^2 ^Algorithm used to predict the sequence of the immunogenic peptide: B for BEPITOPE otherwise PEPOP. ^3 ^The reference segment chosen is the longest (L: longest) or the segment at the top (T) of the list of all the segments ranked according to the five characteristics indicated in the text. ^4 ^The method of extension chosen is either elongation with the flanking protein sequence (FPS); addition of the nearest neighbor (NN) segment(s) regardless of the sense of the segment added: if the segment is in the unnatural sense, i.e., from the C-terminus to the N-terminus, it is synthesized using D-amino acids (NNd); or manual design (MD). ^5 ^The area of extension is the cluster top of the list (TC: top cluster) of the clusters ranked according to the five characteristics (see the text), or the cluster containing the selected reference segment (CC: containing cluster), or a user selected cluster (SC: selected cluster), or none when the reference segment is the longest and the method is the FPS. ^6 ^Positive reactivities in the indirect ELISA are values over 0.2, and strong reactivities are values over 1.0. Control experiments done by using the preimmune serum gave values ranging from 0.0020 to 0.0028. The given values correspond to specific absorbances (A450 nm immune serum – A450 nm preimmune serum).

#### B- Experimental evaluation of the immunogenicity of designed peptides

To prove the capacity of PEPOP to successfully predict peptides that are able to induce Abs able to recognize the cognate protein, several peptides were designed from the 3D structure of three model Ags and chemically synthesized, then rabbits were immunized with these peptides (conjugated to a carrier protein), and the reactivity of the resulting Abs with the cognate Ags was measured. From the 3D structures of the PTP domain of IA-2, the MPO-like domain of TPO, and IL8, PEPOP was run to predict clusters of surface accessible residues and to design peptides from them. Several of the possible methods to construct peptides *in silico *were used although not all could be tested on the three Ags, thus precluding a strict comparison of methods. Nevertheless, five peptides (peptides #4–8) were designed from the IA-2 model, four from the TPO model (peptides #13–16), and two from the 3D structure of IL8 (peptides #17 and #18). All but three of the peptides predicted by PEPOP have the longest identified segment as reference segment. The other three peptides started from the top-ranked segment either of the top-ranked cluster (peptide #15) or of a user-selected cluster (peptide #8 and #18). To predict the peptides, the simplest method [the flanking protein sequence (FPS) process] was first tested (peptides #4, #8, #13, #17, and #18 in Table 3). Then, the ability of the software to predict immunogenic peptides corresponding to a discontinuous epitope of the protein was experimented with the use of the NN method to design peptides #5, #6, #14 and #15. The design process is illustrated in Figure 3. Some other peptides were designed "manually", i.e., by using user-defined information (peptides #7 and #16 in Table 3). To evaluate the method, seven peptides (peptides #1, #2, #3, #9, #10, #11, and #12) were predicted from the amino acid sequence of TPO and IA-2 by using the standard algorithm BEPITOPE [11]. This method predicts peptides from the protein sequence, the corresponding epitopes thus being continuous.

Synthetic peptides were prepared according to the designed sequences, then coupled to KLH and used to immunize rabbits. To satisfy the predictive goal, anti-peptide polyclonal Abs must recognize the predicted peptide and cross-react with the cognate protein. Table 3 presents the reactivity with the cognate Ag of the Abs obtained by immunization with the different peptides (all Abs reacted strongly with the cognate peptide; results not shown). The best results (highest ELISA reactivity with the Ag) were obtained with peptides designed by the FPS method since four out of five such peptides led to the production of Abs that reacted strongly with the protein Ag (peptides #4 and #8 with IA-2, peptide #13 with TPO, and peptide #18 with IL8). Only peptide #17 failed to recognize the IL8 Ag. Peptides designed by the FPS method of PEPOP led more frequently to strongly reactive sera than peptides selected according to the predictions of BEPITOPE: two control peptides (#1 and #9) induced an Ab response of the same quality (as measured by ELISA), whereas the five other control peptides (peptide #2; #3, #10, and #12) gave rise to medium-range reactivities (Table 3), and peptide #11 did not lead to any significant Ab response. The two peptides (#7 and #16) that were "manually designed" as well as the retro-inverso peptide (#6) did not lead to any significant Ab response. Two peptides out of the four designed by using the NN method of PEPOP gave intermediate cross-reactivity with the cognate Ag reactivity (peptides #14 and #15 on TPO). In summary, the simple FPS method (which provides continuous sequences) proved to be very efficient to yield peptides which, when conveniently coupled to a suitable carrier, induced a strong Ab response against the corresponding protein Ag. Except in a few cases, more sophisticated design methods (NN, NNd, and MD) that tried to reconstitute discontinuous epitopes failed to propose peptides which had useful immunogenic properties. As compared with peptides designed from the standard method (BEPITOPE), PEPOP performed equally well or even better.

#### C- Use of the PEPOP algorithm for sandwich immunoassay design

**Figure 4:**
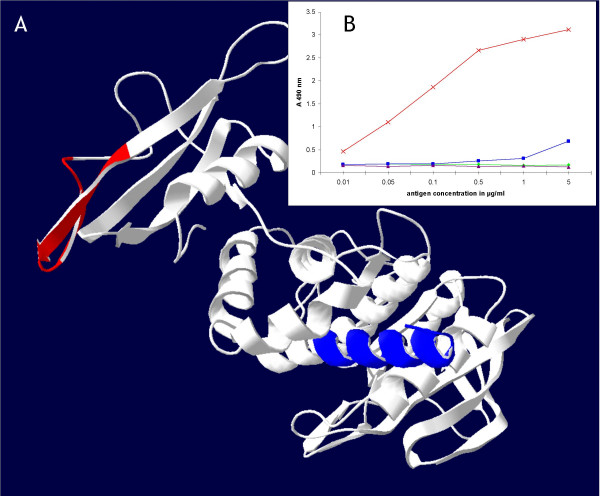
**Sandwich immunoassay design for IA-2**. **(A) **3D view. Peptide #1 (in red) and peptide #4 (in blue) are represented on the 3D structure of IA-2. **(B) **Graphic representation. The graph indicates the reactivity of the polyclonal (Abs) with IA-2, in a sandwich-type ELISA format. The Ag, captured by the immobilized Ab, is detected by a biotinylated Ab, followed by addition of streptavidin-peroxidase. Reactivity of×, anti-peptide #4 with IA-2, detected by anti-peptide #1; ▲, anti-peptide #4 with TPO, detected by anti-peptide #1; ■, anti-peptide #15 with IA-2, detected by anti-peptide #1; ◆, anti-peptide #15 with TPO, detected by anti-peptide #1.

Since PEPOP is able to predict immunogenic peptides localized on the surface of the 3D structure of a target protein, it could conceivably be used to select two candidate peptides that are structurally appropriately separated in the 3D model such that they would *a priori *generate Abs able to react with the protein in a sandwich assay (i.e., an assay in which the protein Ag, in solution, is captured by two different Abs). As an example of such an application of PEPOP, Figure 4A shows the localization of peptide #1 and peptide #4 on the 3D structure of IA-2. Peptide #1 corresponds to an α-helical part of the PTP domain of IA-2, whereas peptide #4 maps to a hairpin in the juxta-membrane domain. These peptides clearly belong to spatially opposite regions on the protein. The resulting anti-peptide Abs were used in a sandwich ELISA to validate their capacity to simultaneously bind the cognate Ag. The results (Figure 4B) demonstrate the possibility to use PEPOP to target specific regions of the protein so as to obtain a pair of Abs able to capture and quantify the protein of interest in solution.

## Discussion

This work was conducted in the context of an abundance of epitope predictive methods based on protein sequence information and a relative scarcity of methods using 3D information. Since the majority of Abs against protein Ags binds to discontinuous sites, predictive methods should take into account structural information and try to guess the identity of discontinuous epitopes. This idea has recently been taken into account by a few groups of investigators [21-24].

PEPOP is a two-purpose algorithm. On the one hand, it allows the identification of clusters of accessible surface residues and segments that might correspond to antigenic epitopes, and on the other hand, it is able to design peptides that can be used to prepare Abs that target the cognate Ag at specific sites on its molecular surface. From the 3D structure of a protein, PEPOP identifies segments composed of one to n continuous surface accessible residues. These segments are clustered according to their spatial vicinity: thus, the clusters are proposed to be putative discontinuous epitopes. Segments and clusters are further used to design putatively immunogenic peptides. Antigenicity is the ability of a protein to bind specifically to the paratope of an Ab. Immunogenicity is the ability of an Ag to induce an immune response in a suitable host. Thus, antigenicity is just a molecular recognition phenomenon, whereas immunogenicity depends on multiple factors extrinsic to the sole Ag [30]. In developing and using PEPOP, we have clearly distinguished these two properties and we show that the outputs of PEPOP could be used in both settings. As concerns antigenicity, one has to keep in mind that epitopes are in essence unpredictable since epitopes are not intrinsic features of proteins but exist only in the context of the Ab that binds to it [31]. Thus, what one generally predicts is at best a putative epitope to which an (undefined) Ab might bind. Our method, as all others in the same vein, is a tool that helps experimentalists to focus on a defined part of a protein for experimentally assessing its real antigenic character by measuring antibody binding to this particular part.

A key step in proposing a new predictive tool is the demonstration of its efficacy [32]. To evaluate the performance of an algorithm to predict antigenicity seems rather easy since it consists of comparing the predictions with known epitopes. The gold standard for comparison is an epitope, as defined by X-ray crystallography of an Ab-Ag complex since this method provides a comprehensive topological view of the epitope. However, it certainly counts as epitope residues, some residues which are not energetically involved in the interaction with antigen (Novotny, 1989), and this may bias the comparison between predicted and epitopes defined by X-ray crystallography. The authors of the three reported predictive algorithms that make use of 3D information used different methods for evaluation of the algorithm performances. Batori *et al*. [22] reported results on a single Ab-Ag model, but they rigorously compared the performances of their software with that of sequence-based algorithms. As expected, their EMT algorithm performed better than conventional methods [22,33]. To assess the performance of their DiscoTope tool, Haste Andersen *et al*. [23] compared its predictions with those obtained by the method of Parker *et al*. [14] and by a surface accessibility calculation. They also reported epitope prediction for a single antigen. The evaluation strategy developed by Kulkarni-Kale *et al*. [24] was similar to the one we undertook. Their rationale for prediction was also very similar to ours. It seems, however, that their CEP algorithm tends to predict much larger antigenic determinants than PEPOP. Authors of the fourth tool, the MEPS server [21], did not present any validation data. The ability of PEPOP to identify putative antigenic regions on proteins was evaluated by comparing 13 structurally defined epitopes on a total of 8 different protein Ags with the clusters calculated by the algorithm. The identified clusters were shown to correspond to a large (but not complete) extent to structurally defined epitopes. In 11 out of the 13 Ab-Ag complexes analyzed, at least 50% of the structural epitope residues were found to be included in a single PEPOP cluster. In the last two Ab-Ag complexes [PDB: 1VFB, 1EY0], the predicted residues almost equally distribute into three clusters, thus yielding the lowest sensitivity numbers. It should be noted that our method being based on detection of exposed residues, residues buried in the structure that may participate to epitopes are missed. CEP, EMT and DiscoTope also make use of the accessibility parameter in their calculations. The comparative assessment of sensitivity, specificity and positive predictive value of PEPOP, CEP, and DiscoTope showed that PEPOP achieves similar or slightly better performances than the other two algorithms.

As regards to immunogenicity, assessing the performance of a tool to predict immunogenic peptides is more difficult because it implies time consuming experiments, notably immunizing animals. As others [34,35], we have made this effort. To demonstrate the ability of PEPOP to successfully predict immunogenic peptides, several peptides were designed from the 3D structure of three model Ags using different methods; they were then chemically synthesized and used to elicit Abs in rabbits. Since all peptides induced a strong anti-peptide antibody response (data not shown) and not all antibodies recognized the cognate protein, it was assumed that antibodies did not cross-react with a denatured antigen. The capacity of the Abs to cross-react with the target Ag was taken as proof of a successful prediction. The FPS method of peptide design was found to be efficient to yield peptides able to induce a strong Ab response against the cognate protein Ag since an 80% success rate was achieved. The FPS method provides a peptide with a sequence made from contiguous residues of the protein, thus the 3D information is lost. However, as compared with peptides designed by using BEPITOPE [18], the FPS method of PEPOP performed better. Both PEPOP and BEPITOPE use the β-turn criteria to predict epitope (assignment in PEPOP, propensity of the antigen sequence in BEPITOPE). PEPOP, however, uses four additional parameters, the length of the segment, its hydrophobicity, its accessibility as well as the occurrence of R, W, Y, and P, that have been found to be overrepresented in protein-protein interfaces [36]. It is possible that this aggregated information might add some value to the prediction simply based on β-turn propensity. Note, however, that the performances of BEPITOPE have recently been surpassed by machine learning approaches [37]. A disappointing observation was that our attempts to design peptides mimicking discontinuous regions of the Ags were not fully successful, in that the corresponding Abs were poorly or not at all cross-reactive with the cognate protein. Cross-reactive antibodies were obtained with several peptides (designed notably using the NN method), but their reactivity was low as compared with FPS-based antibodies. Chemically mimicking discontinuous protein surfaces have been a long-standing scientific challenge [38]. We are nevertheless confident that this could be amenable since many mimotopes have been identified that are considered as low molecular weight functional replicas of discontinuous epitopes [39]. As the PEPOP software has only recently been developed, many parameters can probably be improved to better predict discontinuous peptides. For example, the scoring and ranking of the segments can be adjusted and the combination of one to five of the characteristics deserves to be thoroughly tested. Although PEPOP has proved its capacity to predict relevant clusters and immunogenic peptides, it still contains a great potential for improvement, particularly if a prediction → experimental validation → optimization loop is implemented. PEPOP is flexible and can be a useful tool for different purposes. For example, in an antigenic use, PEPOP can help to map an epitope by building up a small virtual peptide library that can then be tested for binding to the Ab; should the Ab recognize a peptide, the epitope is localized. PEPOP has also the potential to predict mimotopes, i.e. peptides without sequence similarity with the Ag sequence which are recognized by the mAb. Mimotopes can have many applications and lead to pharmacological target identification (interesting for drug design or guided docking), to protein engineering, vaccine design, identification of protein function, etc. As concerns immunogenicity, PEPOP can be used to target a specific region of a protein or to obtain Abs that capture the Ag. The need for mAbs to characterize proteins identified by large-scale proteomic studies is ever increasing. Since the protein itself is not always available, the interest in developing a method using peptides as surrogate Ags may have great potential. An interesting feature of the PEPOP algorithm is its possibility to propose putative immunogenic peptides that could yield Abs suitable for a sandwich capture assay of any protein Ag that can be modeled. With the advent of large-scale proteomic studies and Ab arrays, there is an increasing need for such immunoassays [40,41]. An ideal pipeline to fit these requirements would rely on novel high-throughput modeling capacities [42] and bioinformatics tools like PEPOP to select peptides so as to obtain in a straightforward way pairs of surface-targeted Abs for developing sandwich assays for diagnostic or discovery purposes.

## Conclusion

PEPOP can identify epitopes at the surface of proteins with accuracy comparable to similar tools available through web interface. Moreover, PEPOP can also be used to design immunogenic peptides from the 3D coordinates of the protein.

## Methods

### PEPOP algorithm

#### Clustering of the accessible surface segments

From the 3D structure of a protein, the solvent accessible surface area is calculated (Figure 1A, step 1). The surface accessibility of amino acids is determined by using DSSP [43] with the default parameters. Segments composed of accessible and contiguous amino acids are then listed (a segment can be constituted by a single amino acid) (Figure 1A, step 2). Each segment is then approximated to a geometric segment represented by three points: the first two points are the Cα of the N-terminal and C-terminal residues of the segment, the third point is calculated as the mid-point between the other two. Hence, a segment is represented by the 3D coordinates (X, Y, Z) of the three characteristic points. A distance matrix is then calculated in which the comparison between two segments produces nine values since a segment is represented by three points. A matrix of the shortest distances is then derived in which the distance between two segments is represented by the lowest value among the nine previously calculated. This matrix is used to cluster the segments (Figure 1A, step 3). The clustering of the segments is performed by Kitsch (from PHYLIP package v3.6) [44]. At this stage, a set of accessible surface segments is identified and segments clustered based on the shortest distance matrix.

#### Scoring the segments

A score is attributed to the segments of a given list (either the segments of a cluster or all the segments identified) for each selected property (segment length, segment accessibility, segment hydrophobicity, occurrence of particular amino acids, and occurrence of residues in β-turns). The length score is the number of amino acids in a segment. The accessibility score is the average accessibility of the amino acids composing the segment (values from DSSP). The hydrophobicity score is the number of hydrophobic amino acids (Y, W, F, L, V, I, C, P, M) in the segment. The particular amino acid score is the number of W, R, Y and P in the segment. The β-turn score is the number of amino acids involved in a β-turn (DSSP assignment).

#### Scoring the clusters

Each cluster is scored for the five properties according to the score of the composing segments. The length score of a cluster is the length of the longest segment of this cluster. The accessibility score is the number of segments of the cluster being part of the first quartile of the most accessible segments. The hydrophobicity score is the sum of the hydrophobic amino acids of the segments contained in the cluster. The particular amino acid score is the sum of W, R, Y, and P of the segments contained in the cluster. The β-turn score is the sum of amino acids implied in a β-turn of the segments contained in the cluster.

#### Ranking the segments or the clusters

The segments or the clusters are ranked for each of the five properties according to the assigned score. The five ranks of a segment or a cluster are summed, and the segments or clusters are finally ranked according to these sums.

#### Methods to design immunogenic peptides

To design a peptide, a "reference segment" is chosen from the set of identified segments and its sequence extended with residues selected by a "method of extension" in a specified "area of extension". The reference segment can be manually selected among all the segments if a particular region of the protein is desired to be targeted and so present in the final peptide. Otherwise, the PEPOP algorithm is devised to automatically select the first ranked segment according to criteria selected among the five physicochemical or structural properties of the amino acids. These parameters were chosen in agreement with different analyses of Ab-Ag and/or protein-protein interactions (among them [45,46]) that have shown that they play a role in or they favorably influence the antigenicity and even the immunogenicity of a protein, or they are favorably associated with the binding between proteins. In the PEPOP software, these five characteristics can be chosen one by one or combined. The segments are ranked according to the chosen characteristics within each segment: the top scored segment is automatically selected as the reference segment. In a second step, the reference segment is elongated to yield a peptide of a suitable molecular size. Three methods of extension have been implemented. The simplest is the extension of both sides of the sequence of the reference segment with the flanking sequence of the cognate protein (called FPS for Flanking Protein Sequence). In the second method (called NN for nearest neighbours), the segments having the shortest distance from the reference segment are searched for by the algorithm. The sequence of the segment nearest to the C-terminus of the reference segment is added C-terminally (Figure 3). If necessary, a further extension is conducted in the same way, from the last segment added until a defined total minimal length of the peptide sequence is reached. The third method is the search for an optimized path between the segments composing a peptide (called ONN for optimized nearest neighbor path). From a set of segments that composes a peptide, all the possible combinations are explored to select the one for which the global distance between all the segments is the shortest. The last element for the design of the peptide is the choice of the area of extension. The elongation of the peptide can take into account either the whole set of accessible segments from the protein or only a previously determined cluster of accessible segments. This cluster can be chosen among all clusters by the user, or it is automatically selected by the algorithm in the same way as the reference segment was automatically selected. Thus, the clusters are ranked according to the same previously described five characteristics that can be combined: accessibility, length, hydrophobicity, number of R, W, Y, P, and number of β-turns. The cluster having the best rank according to the selected parameters is selected as the area of extension.

### Implementation details

PEPOP has been implemented on a Linux server (Dell PE2250 virtualized server with the Mandriva 2007 OS distribution) running the Apache web server version 2.0. The algorithm has been implemented in object oriented PHP (version 5), which allows the simultaneous development of the web interface. The segments and clusters identified by PEPOP can be directly visualized on the 3D structure of the Ag thanks to the plug-in Chime [47]. Further development will allow the prediction and display of several peptides at the same time.

### Comparison of the PEPOP performances with that of other available epitope prediction tools

The performances of PEPOP were compared with the two other available epitope tools, i. e. DiscoTope and CEP by calculating specificity, sensibility, and positive predictive value derived from a two-by-two contingency table [48,49]:

$$\begin{array}{ccc} Sp=\frac{TN}{\left(TN+FP\right)} & Se=\frac{TP}{\left(TP+FN\right)} & PPV=\frac{TP}{\left(TP+FP\right)} \end{array}$$

where Sp is the specificity, Se the sensibility, PPV the Positive Predictive Value, and TN the number of amino acids not predicted and actually not part of the epitope, TP the number of predicted amino acids that are actually part of the epitope, FP the number of predicted amino acids not part of the epitope, and FN the number of amino acids not predicted but part of the epitope. Therefore, the specificity evaluates the capacity of the tool to exclude those amino acids that are not part of the epitope, and the sensibility measures the capacity of the tool to identify the amino acids of the epitope. The positive predictive value is the proportion of predicted amino acids that are truly part of the epitope.

Note that the outputs of the three epitope prediction tools are different. DiscoTope yields a single prediction, identifying along the protein sequence the amino acids that might belong to an epitope. Instead, PEPOP and CEP suggest several potential epitopes. By grouping the segments, PEPOP identifies a few exclusive clusters, i.e., the potential epitopes are not overlapping. CEP proposed several CE (conformational epitope) that may or may not be partially overlapping. For each of the 13 epitope predictions, the Sp, Se, and PPV were calculated on the single proposition of DiscoTope, and on the cluster (for PEPOP) and the CE (for CEP) giving the best values.

### 3D structures of IA-2, TPO, and IL8

The intracytoplasmic part of insulinoma associated antigen 2 (IA-2) ([Swiss-Prot: Q16849] residues 601–979) is composed of two domains: the juxta-membrane domain (residues 601–690) and the PTP domain (residues 691–979). A theoretical model of the PTP domain of IA-2 was previously published by Dromey *et al*. [50] but was not made available in the PDB [51]. Consequently, a new theoretical model was calculated (Moreau, Valera *et al*., in preparation) to determine the 3D coordinates of the structure. The human thyroïd peroxidase (TPO) [Swiss-Prot: P07202] contains a large extracytoplasmic domain (residues 15–846) composed of four domains: a structurally undefined domain (residues 15–140), a MPO-like domain (141–740), a CCP-like domain (residues 741–795), and an EGF-like domain (residues 796–839). A theoretical model of the MPO-like domain has been reported [52] and was made available to us. The interleukin-8 (IL8) [Swiss-Prot: P10145] is composed of a single domain of about 70 amino acids, depending on the variant. Its 3D structure is available in PDB [PDB: 3IL8].

### Peptide predictions

The PEPOP predictions were made with the 3D structures of the PTP domain of IA-2, the MPO-like domain of TPO and the entire IL8. BEPITOPE was used with the default parameters on the Pellequer's TURN33 addition method. The predicted epitopes proposed were ranked according to the average value. The overlapping regions are considered as a unique predicted epitope. The sequences used were the intracytoplasmic part of IA-2, the extracytoplasmic part of TPO, and the entire sequence of IL8.

### Peptide synthesis and purification

Peptides were prepared by Fmoc solid-phase synthesis using an AMS 422 robot or a MultiPep synthesizer (INTAVIS AG, Germany). Peptides were tagged by adding the tripeptide Cys-Tyr-Gly N- or C- terminally to the target sequence to facilitate monitoring at 280 nm during purification and to provide a thiol handle for coupling to a protein carrier. The standard synthesis protocol [53] was used throughout. Mass spectrometry (MALDI-Tof Voyager DE, Applied Biosystems) was used to confirm the identity of the synthetic peptide with the target sequence. Peptides were coupled to keyhole limpet hemocyanin (KLH) by using the heterobifunctional coupling reagent sulfo-SMCC, according to the manufacturer's instructions.

### Immunizations

New Zealand white rabbits (Centre Lago, France) were immunized three times via the intradermic route at 14-day intervals using 50 μg of Ag (KLH-coupled peptides) and Freund's complete or incomplete adjuvant. Rabbits were bled 10 days after the third immunization and the serum titer measured by ELISA. The rabbits were boosted twice subcutaneously at 21-day intervals with 50 μg of Ag prior to terminal blood collection (by cardiac puncture).

### Ab reactivity with the cognate protein

The entire IL8 (recombinant protein produced in *E. coli*) was purchased from Peprotech.

Extracytoplasmic TPO was purchased from HyTest Ltd. The intracytoplasmic part of IA-2 (residues 601–979) was produced in the baculovirus-insect cell system in our laboratory.

For indirect ELISA, maxisorp 96-well plates were coated with 2 μg/ml of Ag in phosphate-buffered saline (PBS) (overnight at 4°C). The plates were washed in PBS containing 0.1% Tween-20 (PBS-T) and blocked with 2% nonfat milk in PBS-T (1 h at 37°C). One hundred microlitres of serial dilutions of the final bleeds (1:5 000 to 1:1 000 000) in 2% nonfat milk PBS-T was added to each well (2 h at 37°C). After three washes in PBS-T, the plates were incubated with a peroxidase-conjugated anti-rabbit Ab (Sigma), diluted 1:3 000 in PBS-T 2% nonfat milk (1 h at 37°C). Plates were washed four times in PBS-T and incubated with OPD. After 20 min at room temperature, the absorbance at 450 nm was measured.

For sandwich ELISA, maxisorp 96-well plates were coated with 2 μg/ml of Protein A purified Ab (overnight at 4°C). After blocking with 3% bovine serum albumin (BSA) in PBS-T (1 h at 37°C), 100 μl of serial dilutions of the Ag (0.01 μg/ml to 5 μg/ml in 3% BSA-PBS-T) was added (2 h at 37°C) to each well. After three washes in PBS-T, the plates were incubated with 2 μg/ml of a biotinylated-conjugated purified polyclonal Ab in 3% BSA-PBS-T (2 h at 37°C). After three washes in PBS-T, the plates were incubated with peroxidase-conjugated streptavidin (Amersham), diluted 1:3 000 in 3% BSA-PBS-T (1 h at 37°C). Plates were washed four times in PBS-T and incubated with OPD substrate for 20 min at room temperature. The reaction was stopped by adding 50 μl of 4 N H_2_SO_4 _and the absorbance at 490 nm was measured. In control experiments, rabbit preimmune sera were tested in the above conditions. The absorbance value noticed for each dilution was subtracted from the absorbance value given by the corresponding dilution of the rabbit immune serum.

## Availability and requirements

PEPOP is a server web based application and is usable as a Sysdiag Service at . This service is plateform independent, fully tested with Windows 2000™, Windows XP™. Programming language: PHP; required the installation of the plug-in Chime (not compatible with all the navigators) to visualize the results in the 3D structure of the protein.

## Authors' contributions

VM and FM developed the concept of the method, VM and CF wrote the computer code and implemented the Web interface. VM performed the predictions for the evaluation tests. For the antigenic evaluation, VM and CF collected the data; VM, CG, FM, and CF analyzed the results. For the immunogenic evaluation, CN synthesized the peptides; DP, SV and NN carried out the immunizations and tested the binding reactivities. CG, VM, DL, and FM analyzed the results. CG, VM, DL and FM participated in writing the manuscript. All authors read and approved the final manuscript.

## References

[B1] Edwards AM, Arrowsmith CH, Christendat D, Dharamsi A, Friesen JD, Greenblatt JF, Vedadi M (2000). Protein production: feeding the crystallographers and NMR spectroscopists. Nat Struct Biol.

[B2] Carter P (1986). Site-directed mutagenesis. Biochem J.

[B3] Szklarz GD, Halpert JR (1997). Use of homology modeling in conjunction with site-directed mutagenesis for analysis of structure-function relationships of mammalian cytochromes P450. Life Sci.

[B4] Frank R (2002). The SPOT-synthesis technique. Synthetic peptide arrays on membrane supports – principles and applications. J Immunol Methods.

[B5] Reineke U, Kramer A, Schneider-Mergener J (1999). Antigen sequence- and library-based mapping of linear and discontinuous protein-protein-interaction sites by spot synthesis. Curr Top Microbiol Immunol.

[B6] Eichler J (2005). Synthetic peptide arrays and peptide combinatorial libraries for the exploration of protein-ligand interactions and the design of protein inhibitors. Comb Chem High Throughput Screen.

[B7] Geysen HM, Barteling SJ, Meloen RH (1985). Small peptides induce antibodies with a sequence and structural requirement for binding antigen comparable to antibodies raised against the native protein. Proc Natl Acad Sci USA.

[B8] Halperin I, Wolfson H, Nussinov R (2003). SiteLight: binding-site prediction using phage display libraries. Protein Sci.

[B9] Moreau V, Granier C, Villard S, Laune D, Molina F (2006). Discontinuous epitope prediction based on mimotope analysis. Bioinformatics.

[B10] Wilson IA, Niman HL, Houghten RA, Cherenson AR, Connolly ML, Lerner RA (1984). The structure of an antigenic determinant in a protein. Cell.

[B11] Pellequer JL, Westhof E, Van Regenmortel MH, Wisdow GB (1994). Epitope prediction from primary structure of proteins. Peptide Antigens: A Practical Approach.

[B12] Kolaskar AS, Tongaonkar PC (1990). A semi-empirical method for prediction of antigenic determinants on protein antigens. FEBS Lett.

[B13] Alix AJ (1999). Predictive estimation of protein linear epitopes by using the program PEOPLE. Vaccine.

[B14] Parker JM, Guo D, Hodges RS (1986). New hydrophilicity scale derived from high-performance liquid chromatography peptide retention data: correlation of predicted surface residues with antigenicity and X-ray-derived accessible sites. Biochemistry.

[B15] Jameson BA, Wolf H (1988). The antigenic index: a novel algorithm for predicting antigenic determinants. Comput Appl Biosci.

[B16] Welling GW, Weijer WJ, van der Zee R, Welling-Wester S (1985). Prediction of sequential antigenic regions in proteins. FEBS Lett.

[B17] Van Regenmortel MH, Pellequer JL (1994). Predicting antigenic determinants in proteins: looking for unidimensional solutions to a three-dimensional problem?. Pept Res.

[B18] Odorico M, Pellequer JL (2003). BEPITOPE: predicting the location of continuous epitopes and patterns in proteins. J Mol Recognit.

[B19] Blythe MJ, Flower DR (2005). Benchmarking B cell epitope prediction: underperformance of existing methods. Protein Sci.

[B20] Moult J (2005). A decade of CASP: progress, bottlenecks and prognosis in protein structure prediction. Curr Opin Struct Biol.

[B21] Castrignano T, De Meo PD, Carrabino D, Orsini M, Floris M, Tramontano A (2007). The MEPS server for identifying protein conformational epitopes. BMC Bioinformatics.

[B22] Batori V, Friis EP, Nielsen H, Roggen EL (2006). An in silico method using an epitope motif database for predicting the location of antigenic determinants on proteins in a structural context. J Mol Recognit.

[B23] Haste Andersen P, Nielsen M, Lund O (2006). Prediction of residues in discontinuous B-cell epitopes using protein 3D structures. Protein Sci.

[B24] Kulkarni-Kale U, Bhosle S, Kolaskar AS (2005). CEP: a conformational epitope prediction server. Nucleic Acids Res.

[B25] Novotny J, Handschumacher M, Haber E, Bruccoleri RE, Carlson WB, Fanning DW, Smith JA, Rose GD (1986). Antigenic determinants in proteins coincide with surface regions accessible to large probes (antibody domains). Proc Natl Acad Sci USA.

[B26] Thornton JM, Edwards MS, Taylor WR, Barlow DJ (1986). Location of 'continuous' antigenic determinants in the protruding regions of proteins. Embo J.

[B27] The Protein DataBase. http://www.rcsb.org/pdb/home/home.do.

[B28] DiscoTope. http://www.cbs.dtu.dk/services/DiscoTope/.

[B29] CEP. http://202.41.70.74:8080/cgi-bin/cep.pl.

[B30] Van Regenmortel MH (2001). Antigenicity and immunogenicity of synthetic peptides. Biologicals.

[B31] Van Regenmortel MH (2004). Reductionism and complexity in molecular biology. Scientists now have the tools to unravel biological and overcome the limitations of reductionism. EMBO Rep.

[B32] Greenbaum JA, Haste Andersen P, Blythe M, Bui HH, Cachau RE, Crowe J, Davies M, Kolaskar AS, Lund O, Morrison S (2007). Towards a consensus on datasets and evaluation metrics for developing B-cell epitope prediction tools. J Mol Recognit.

[B33] Roggen EL (2006). Recent developments with B-cell epitope identification for predictive studies. Journal of Immunotoxicology.

[B34] Di Giambattista M, Branckaert T, Hougardy V, Kemball-Cook G, Laub R (2007). In silico prediction of FVIII epitopes recognised by natural autoantibodies in polyvalent immunoglobulin concentrates. Mol Immunol.

[B35] Renukaradhya GJ, Mitra-Kaushik S, Sinnathamby G, Rajasekhar M, Shaila MS (2002). Mapping of B-cell epitopes of hemagglutinin protein of rinderpest virus. Virology.

[B36] Young L, Jernigan RL, Covell DG (1994). A role for surface hydrophobicity in protein-protein recognition. Protein Sci.

[B37] Sollner J, Mayer B (2006). Machine learning approaches for prediction of linear B-cell epitopes on proteins. J Mol Recognit.

[B38] Reineke U, Sabat R, Misselwitz R, Welfle H, Volk HD, Schneider-Mergener J (1999). A synthetic mimic of a discontinuous binding site on interleukin-10. Nat Biotechnol.

[B39] Meloen RH, Puijk WC, Slootstra JW (2000). Mimotopes: realization of an unlikely concept. J Mol Recognit.

[B40] Kusnezow W, Hoheisel JD (2002). Antibody microarrays: promises and problems. Biotechniques.

[B41] Michaud GA, Salcius M, Zhou F, Bangham R, Bonin J, Guo H, Snyder M, Predki PF, Schweitzer BI (2003). Analyzing antibody specificity with whole proteome microarrays. Nat Biotechnol.

[B42] Pieper U, Eswar N, Davis FP, Braberg H, Madhusudhan MS, Rossi A, Marti-Renom M, Karchin R, Webb BM, Eramian D (2006). MODBASE: a database of annotated comparative protein structure models and associated resources. Nucleic Acids Res.

[B43] Kabsch W, Sander C (1983). Dictionary of protein secondary structure: pattern recognition of hydrogen-bonded and geometrical features. Biopolymers.

[B44] Felsenstein J (1989). PHYLIP – Phylogeny Inference Package (Version 3.2). Cladistics.

[B45] Bogan AA, Thorn KS (1998). Anatomy of hot spots in protein interfaces. J Mol Biol.

[B46] Chakrabarti P, Janin J (2002). Dissecting protein-protein recognition sites. Proteins.

[B47] Chime. http://www.mdl.com/.

[B48] Pretty IA, Maupome G (2004). A closer look at diagnosis in clinical dental practice: part 2. Using predictive values and receiver operating characteristics in assessing diagnostic accuracy. J Can Dent Assoc.

[B49] Swets JA (1988). Measuring the accuracy of diagnostic systems. Science.

[B50] Dromey JA, Weenink SM, Peters GH, Endl J, Tighe PJ, Todd I, Christie MR (2004). Mapping of epitopes for autoantibodies to the type 1 diabetes autoantigen IA-2 by peptide phage display and molecular modeling: overlap of antibody and T cell determinants. J Immunol.

[B51] Sussman JL, Lin D, Jiang J, Manning NO, Prilusky J, Ritter O, Abola EE (1998). Protein Data Bank (PDB): database of three-dimensional structural information of biological macromolecules. Acta Crystallogr D Biol Crystallogr.

[B52] Hobby P, Gardas A, Radomski R, McGregor AM, Banga JP, Sutton BJ (2000). Identification of an immunodominant region recognized by human autoantibodies in a three-dimensional model of thyroid peroxidase. Endocrinology.

[B53] Laune D, Molina F, Ferrieres G, Villard S, Bes C, Rieunier F, Chardes T, Granier C (2002). Application of the Spot method to the identification of peptides and amino acids from the antibody paratope that contribute to antigen binding. J Immunol Methods.

[B54] Amit AG, Mariuzza RA, Phillips SE, Poljak RJ (1985). Three-dimensional structure of an antigen-antibody complex at 6 A resolution. Nature.

[B55] Chitarra V, Alzari PM, Bentley GA, Bhat TN, Eisele JL, Houdusse A, Lescar J, Souchon H, Poljak RJ (1993). Three-dimensional structure of a heteroclitic antigen-antibody cross-reaction complex. Proc Natl Acad Sci USA.

[B56] Braden BC, Souchon H, Eisele JL, Bentley GA, Bhat TN, Navaza J, Poljak RJ (1994). Three-dimensional structures of the free and the antigen-complexed Fab from monoclonal anti-lysozyme antibody D44.1. J Mol Biol.

[B57] Sheriff S, Silverton EW, Padlan EA, Cohen GH, Smith-Gill SJ, Finzel BC, Davies DR (1987). Three-dimensional structure of an antibody-antigen complex. Proc Natl Acad Sci USA.

[B58] Kondo H, Shiroishi M, Matsushima M, Tsumoto K, Kumagai I (1999). Crystal structure of anti-Hen egg white lysozyme antibody (HyHEL-10) Fv-antigen complex. Local structural changes in the protein antigen and water-mediated interactions of Fv-antigen and light chain-heavy chain interfaces. J Biol Chem.

[B59] Li Y, Li H, Smith-Gill SJ, Mariuzza RA (2000). Three-dimensional structures of the free and antigen-bound Fab from monoclonal antilysozyme antibody HyHEL-63. Biochemistry.

[B60] Prasad L, Waygood EB, Lee JS, Delbaere LT (1998). The 2.5 A resolution structure of the jel42 Fab fragment/HPr complex. J Mol Biol.

[B61] Huang M, Syed R, Stura EA, Stone MJ, Stefanko RS, Ruf W, Edgington TS, Wilson IA (1998). The mechanism of an inhibitory antibody on TF-initiated blood coagulation revealed by the crystal structures of human tissue factor, Fab 5G9 and TF.G9 complex. J Mol Biol.

[B62] Fleury D, Daniels RS, Skehel JJ, Knossow M, Bizebard T (2000). Structural evidence for recognition of a single epitope by two distinct antibodies. Proteins.

[B63] Spiegel J, P C, Jacquemin M, Saint-Remy JM, Stoddard BL, Pratt KP (2001). Structure of a factor VIII C2 domain-immunoglobulin G4kappa Fab complex: identification of an inhibitory antibody epitope on the surface of factor VIII. Blood.

[B64] Tulip WR, Varghese JN, Webster RG, Laver WG, Colman PM (1992). Crystal structures of two mutant neuraminidase-antibody complexes with amino acid substitutions in the interface. J Mol Biol.

[B65] Lescar J, Pellegrini M, Souchon H, Tello D, Poljak RJ, Peterson N, Greene M, Alzari PM (1995). Crystal structure of a cross-reaction complex between Fab F9.13.7 and guinea fowl lysozyme. J Biol Chem.

[B66] Bossart-Whitaker P, Chang CY, Novotny J, Benjamin DC, Sheriff S (1995). The crystal structure of the antibody N10-staphylococcal nuclease complex at 2.9 A resolution. J Mol Biol.

